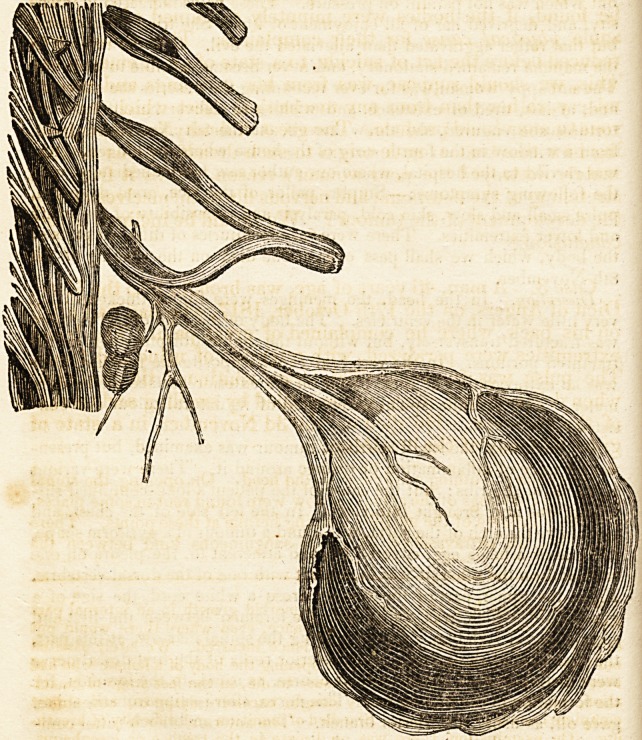# Dr. Ollivier on Diseases of the Spinal Marrow

**Published:** 1824-06-01

**Authors:** 


### MEDICO-CHIRURGICAL

### REVIEW.

Vol. V.] Analytical Series. [No. 17.

" Nec tibi quid liceat sed quid fecisse decebit
" Occurrat, mentemque domat respectus honesti."

Vol. I.] JUNE 1, 1824. [No. 1.

[NEW SERIES.]

### I.

### DISEASES OF THE SPINAL MARROW.

*De la* Moelle Epiniere *et de ses Maladies; Ouvrage couronné par la Societé Royale de Medecine de Marseilles, dans sa Séance Publique du* 23 *Octobre*, 1823. Par C. P. Ollivier, M. D. &c. &c. Octavo, pp. 404, two lythographic Plates. Paris, 1824.

It has been acknowledged for several years past, that affections of the spinal marrow and its membranes are much more common than was formerly suspected—and that these affections often produce disturbance of function in various important organs, in a very occult manner with respect to the original seat of the disease. The difficulty of examining the spine, in our *post mortem* researches, has greatly contributed to keep its pathology obscured—for, except in hospital practice, it is almost impossible to have a satisfactory view of this important portion of the nervous system—and even in hospitals, we fear the time and labour required for spinal dissections, operate powerfully in preventing the toilsome investigation. Even the simple anatomy of the spine is far less familiar to the eye of the practitioner, than that of other parts of the body. He sees the spine two or three times demonstrated or lectured on, during his studies; but, afterwards, in the common routine of practice, he loses all intimate acquaintance with the part, and becomes incapable of appreciating the less obtrusive deviations from its natural structure, should he, by accident, take the trouble of laying open the vertebral canal in search of the seat of diseases. On all these accounts, we must certainly consider ourselves greatly indebted to any individual who imposes on himself the task of investigating the pathology of the spine, even although the first attempts should be imperfect and unsatisfactory.

Dr. Ollivier's work has been crowned with the prize by the Royal Society of Medicine of Marseilles, and thus brings a respectable passport in its title-page. The body of the work evinces talent, labour, zeal, and considerable research—we, therefore, deem it worthy of a very full analysis in our pages.

The volume is divided into three parts; viz. the Anatomy, Physiology, and Pathology of the Spine. The anatomy we must pass over entirely, as that must be learnt in the dissecting-room; and the physiology of the organ we must be very brief with, in order that we may be able to dedicate more space to its diseases.

### CAP. I.—FUNCTIONS OF THE MEDULLA SPINALIS.

Hippocrates observed, that lesions of the spinal chord caused paralysis of the parts situated below the point of lesion, and this was confirmed by the experiments of Galen. It is now well known, that the spinal marrow exercises a direct influence on the production of sensation and motion in the trunk and members. It is, therefore, the *agent* of voluntary action, though, in this respect, it is dependant on the brain, notwithstanding some facts which seem to indicate its independence. "These facts only prove that the influence of the brain on certain actions of life, is so much the less marked in proportion as the animal exhibits a less perfect organization." But the influence of the spinal marrow is not confined to the production of sensation and muscular motion—it is extended to certain vital functions, for example, circulation and respiration. Although Haller attributed the action of the heart to its own innate irritability, he afterwards confessed, that the spinal marrow exercised a special influence on that action; a strange contradiction in a man like Haller! Experiments, however, have proved that the action of the heart is not entirely and immediately dependent on the spinal marrow, as that action has continued when the spinal marrow was removed, and fœtuses have been born without a spinal marrow at all. Nevertheless, the fact is equally certain, that where and while the spinal marrow does exist, it influences decidedly the action of the heart. This is proved beyond all doubt by innumerable pathological facts.

The influence of this organ on respiration is much more direct and obvious—in fact, this function is entirely under the influence of the spinal marrow, at least, in mammiferous animals, who die suffocated as soon as the par vagum (which originates in the upper portion of spinal marrow) is cut. "In respect then to respiration and circulation, we may consider the spinal marrow as an isolated centre of vitality, independent of the brain." 65. Many authors have, also, considered the spinal marrow as influencing several other functions of the interior life, through the medium of the great sympathetic nerve, which has, according to Legallois, its roots in the spinal marrow. This idea is strengthened by the observations of Weber on the great sympathetic. He remarked, that the development of this nerve was always in proportion to that of the spinal marrow, in the various classes of animals which he has examined. In fine, our author thinks it highly probable, that the spinal marrow, directly or indirectly, presides over the whole of the interior functions of the thorax and abdomen, seeing that the par vagum and great sympathetic have their sources in this prolongation of the brain. It is well known, that the experiments of our ingenious countryman, Mr. Brodie, led him to conclude that the power of generating animal heat resided in the brain. About four years ago, M. Chossat repeated Mr. Brodie's experiments, and the results led him to conclude, that this calorific power was shared by the spinal marrow. In these experiments, the section of the medulla spinalis at the occipital foramen, or, indeed, between any of the cervical vertebræ, effected an equal depression of temperature, under artificial respiration, as destruction of the brain itself.

In this place, Dr. Ollivier alludes to the recent experiments of M. Fleurens on the nervous system, of which we took some notice in the 15th Number of this Journal, page 705-6. That gentleman and some others, especially Fodera, Magendie, and Rolando, have ascertained, or think they have ascertained, by experiments on animals, that the property of nervous sensibility, or, as a cotemporary terms it, *impressibility*, is limited to the corpora quadrigemina, medulla oblongata, spinal cord, and nerves—that the integrity of the optic thalami is not essential to the contractility of the iris—that the senses of sight and sound reside in the cerebral lobes—and that *there* all other sensations acquire distinctness and durability—that the spinal cord combines the muscular contractions so as to produce motion in the joints—and, finally, that the cerebellum regulates these movements, and unites them so as to constitute the actions of standing and loco-motion.

On all sides it is admitted, that in the spinal marrow are the foci of feeling and motion; and the experiments of Bell and

Magendie lead us to believe, that in the *posterior* roots of the spinal nerves resides the power of sensation, or, at least, of transmitting impressions to the sensorium; while the *anterior* roots preside over voluntary motion. Such is the succinct sketch which we have traced of the physiology of the spinal cord, and we believe it comprehends most of what is known on the subject.

### CAP. II.—DISEASES OF THE SPINAL MARROW.

It is acknowledged that diseases of this part of our frame are much less common than those of most other parts of the body —infinitely less numerous than those of the brain, for example —and this is *one cause* why they are less known than encephalic affections. Another reason is, the obscurity of their symptoms—and a third, the difficulty of *post mortem* examinations. In tracing the diseases of the spinal marrow, it is proper to begin with original malformations—and here our author acknowledges his obligations to the lectures of M. Beclard on monstrosities.

§ 1. *Absence of the Spinal Marrow.*—No examples are on record of the total want of a spinal marrow, the other parts of the nervous system being entire. When it is wanting the brain is always wanting, but not the converse of this; for there have been numerous acephalous monsters where the spinal marrow was entire. There are many instances recorded of want of both brain and spinal marrow; but only one (Philosophical Transactions, 1793) where the whole nervous system was deficient. It is curious that some of these monsters, without either brain or spinal marrow, have been born alive, and lived many hours, taking nourishment, and shewing signs of sensibility. In the amyelencephalic monsters, (those defective in brain and spinal marrow both) the place of these organs is supplied by membranous pouches or canals filled with a yellowish and viscid liquid, which is generally discharged by rupture of these pouches or canals during parturition. The origins of the nerves present various appearances—sometimes a little eminence or tubercle opposite the intervertebral foramina—sometimes only a few filaments rising from the membrane lining the theca vertebralis. It may be remarked here, that there is not a real, but only an apparent want of brain and spinal marrow in these instances of monstrosity. These parts, in the first rudiments of the embryo, are in a liquid state, and continue so, having never taken on the regular process of organization. It is this liquid which supplies the place and performs the functions of the brain and spinal marrow, till the membranes inclosing it burst, before or after parturition, when the fœtus or infant dies.

§ 2. *Atelomyelia,* or Imperfection of the Spinal Marrow.*—There are several malformations or imperfect organizations of the spinal marrow, as deformities about the upper portion, when the brain is wanting—a division of the medulla spinalis into two separate parts, by a greater or less interval—bifurcations—the existence of a canal in its centre—congenital dropsy. We shall only notice the last of these vices of organization.

*Congenital Hydrorachis.*—This is characterized by one or more tumours, in one or more points of the spine. There are but a very few instances on record where these tumours became developed some years after birth, and consecutively to dropsy of the theca vertebralis. One of the most remarkable is related by Morgagni. The rarity of this phenomenon is easily accounted for, when we consider that the formation of the external tumour generally depends on spina bifida, and that any opening in the bony structure can only take place at a very early period of its ossification. These tumours are of various forms and sizes—sometimes, indeed generally, very circumscribed—but, at others, occupying a great part, or even the whole of the spinal column. Sometimes the tumour is transparent; but more commonly opake, and the colour of the integuments unchanged. The most frequent site of the tumour is in the lumbar region. Occasionally, though more rarely, the tumour has been seen in the region of the sacrum, of which an instance is cited from Vrolik. When there are several tumours, if one be pressed it causes a swelling of the others. If there be hydrocephalus, pressure on the head will distend the hydrorachis—and pressure on this last will, at any time, cause fainting and all the phenomena attendant on cerebral compression, by forcing the fluid up on the brain. The envelopes of these tumours are composed of skin, (which is sometimes nearly transparent and at others entirely wanting) dura mater, tunica arachnoidea, and pia mater. There is, of course, in all cases of this kind, an imperfection in the bony canal. The fluid of hydrorachis is analogous to that of other dropsies—especially of hydrocephalus, with which it is often complicated. The quantity is various, and generally augments with age if the patient lives. In hydrorachis, the spinal

* α privative, τελος perfection, and μυελος medulla.

marrow presents various appearances of malformation; but, sometimes, little or no alteration from the natural structure. There are, also, very often vices of organization in other parts of the body, as transpositions of the viscera, extroversion of the bladder, imperforation of the rectum, &c. This disease, however, does not appear to have any influence on the fœtal life; but, after birth, it generally causes death, at a longer or shorter period from the commencement of its separate existence.

In general, the higher up and larger the tumour, the sooner it proves fatal. When life is prolonged, we usually find the children thus affected feeble, languid, and emaciated—occasionally paralytic. There are, however, some curious exceptions, where boys and girls have grown up with the disease, and appeared to be well nurtured. In general, the tumour augments gradually, sometimes bursting spontaneously, and causing death in the midst of convulsions. There has been one or two instances of cure. Bonn relates the case of a child who lived ten, and Warner the case of a young man who lived twenty years. Camper speaks of another case where the age of 30 years was attained. It is said, that there is now in London a woman, 29 years of age, with hydrorachis. In her, the tumour has attained the size of a man's head, and from its surface there is a slight oozing of liquid. She enjoys good health.

*Treatment.* Death has generally been the result of puncturing the tumour. There are, however, a few exceptions to the contrary. Sir Astley Cooper is well known to have been successful in one case.* Terris's case of cure was by spontaneous rupture of the tumour. In the greater number of those

---

* This eminent surgeon lays down two modes of treatment, one palliative, the other radical. The first consists in treating the case as a hernia, and applying a truss to prevent its descent. The second mode is to procure adhesion of the sides of the sac, so as to close the opening from the spine, and stop the disease altogether. The first is attended with no risk, as the truss forms an artificial vertebra, when the natural one is defective. The truss must be continued through life. On the other hand, the cure by adhesion exposes the patient to much constitutional irritation; but, if successful, it leaves him without apprehension of the future return of the disease. The child cured by Sir Astley in this way, was a specimen of fine health. Our excellent author states, that there are many cases which do not admit of cure. These are as follows:—"If the tumour is connected with an unnatural enlargement of the head, hydrocephalus internus is conjoined with spina bifida, and the water will accumulate in the ventricles, if the tumour in the loins is attempted either to be palliated or radically cured." Again, "If the lower extremities are paralytic, or the fæces and urine are discharged involuntarily, there is no hope of relief." The radical cure consists in puncturing the tumour with a needle as often as the fluid accumulates.

who have had a puncture made, inflammation spread up along the membranes, and destroyed life. Our author thinks that our curative efforts ought to be bounded to the attempt to procure absorption of the fluid, without making any strong pressure on the tumour. When the integuments are not very thin, he recommends the application of blisters, and the actual cautery in the vicinity of the tumour, together with frictions, baths, and, in some cases, gentle pressure cautiously applied. Where there is hydrocephalus, of course this disease is to be attended to particularly.

*Atrophy of the Spinal Marrow.* There are but a very few cases on record of this disease. Bonetus relates two. In one case, where the individual had been subject to convulsions for twelve years, he found the spinal marrow sensibly diminished in size, the vertebral canal containing at the same time a considerable quantity of serosity, by the pressure of which, he conceived, the med. spin. was atrophied. In the other instance there was also a serous effusion, and the patient had been paralytic. Morgagni has remarked, that he has sometimes found the spinal marrow wasted in cases of long-continued hemiplegia. Our author himself, in his researches on the dead body, has twice observed a considerable diminution of volume in the medulla spinalis. One was the case of an idiot, who died at the age of 20, in a complete state of marasmus, especially of the lower extremities, which were also greatly contracted, the legs on the thighs and the thighs on the trunk. There was much effusion into the cavity of the arachnoid of the spine, and the vessels were immensely gorged with blood. The spinal marrow was reduced to about one half of its natural size. There was nothing remarkable in the brain. Our author has observed that in most aged persons there is a wasting, more or less considerable, of the spinal marrow, which phenomenon, he thinks, may, in part at least, account for the unsteady gait of old people.

Our readers are aware that M. Magendie, or rather M. Rullier, has related the case of a gentleman who preserved sensation and motion in the lower extremities, although a portion of the spinal marrow was entirely disorganized and wasted away. The following case is of a similar nature. It was communicated to Dr. Ollivier by M. Van de Keere, *Interne á l'Hôpital des Enfans Malades.*

*Case.* Towards the close of 1820, a child, about eight years of age, with all the symptoms of a scrofulous constitution, died in a state of marasmus. For a long time previously, it had laboured under caries of the spine, accompanied by continual and severe head-ache. Motion and sensation continued in the lower extremities till the last.

*Dissection*. On opening the spinal canal there was a want of continuity in the medulla spinalis, between the ninth dorsal and first lumbar vertebræ—that is, for a space of nearly four inches. In this space the spinal envelopes were flattened together, but not materially changed in structure. When laid open there was no trace of spinal marrow in this portion, it having been compressed and annihilated by an acute angle of the spine, formed by an approximation of the ninth dorsal to the first lumbar vertebræ, with an interruption of the vertebral canal.

"*Dans le point où existait la destruction de la moelle, le rachis formait un angle tres-aigu resultant du rapprochement de la neuvième vertebre dorsale avec la première lombaire; il y avait interruption de son canal.*" P. 145.

There was extensive caries and tubercular disorganization about this part of the spine. The lower extremities were greatly wasted.

§ 3. *Wounds and Contusions of the Spinal Marrow*. These wounds are generally punctures or rents. When the vertebral column is fractured or luxated, (which is almost invariably attended with fracture,*) the spinal marrow may, or rather must be injured. It must be very rare, from the nature of the parts, that the spinal marrow can be simply wounded without fracture or other serious injury of the osseous fabric. In contusions of the spinal marrow, where the pia mater is not ruptured, there is often nothing unnatural to be seen in the medulla itself—sometimes, however, we find a small clot of blood in its centre. In

* The case related by Mr. Howship in the New Medical and Physical Journal for December 1812, does not, we imagine, form an unexceptionable exception to this. The man was a bricklayer, who fell from a high scaffold, and when conveyed to an hospital, it was ascertained "that he had suffered subluxation outwards of the eighth dorsal vertebra." The lower extremities were completely paralytic. There was protuberance of the bone, and after several unavailing efforts, "the bone, on a sudden, returned into its natural situation, with a noise audible to the bystanders." Retention of urine now came on, and in a fortnight incontinence of the same, with involuntary discharge of the fæces. The water was thick, bloody, and loaded with mucous and calculous matters. In the course of the third week there were some indications of returning sensation in the thighs, and he complained of tingling pains and pricking sensations. At the close of the third week mortification took place near the anus, and afterwards the penis sloughed. Vomitings now came on; his speech faltered; he grew comatose; had slight convulsions; and died at the period of five weeks from the accident.

As *post mortem* examination was not allowed, it cannot be said positively that this was a luxation or subluxation without fracture.

violent contusions of the spine we generally find the pia mater ruptured, and an effusion into the theca vertebralis. When the medulla is cut across by a ball or other substance, death is generally very quick, and in proportion as the accident is high up in the spine. A curious example, and a melancholy one too, is related by Petit. A little boy, of six or seven years of age, was lifted up by the head in play, while in the shop of a neighbour. A dislocation was the consequence, and he died on the spot! At this moment the father of the child came in, and, in the transport of his rage, struck the unintentional murderer of the child with a cleaver which he held in his hand. The cleaver penetrated between the first and second cervical vertebræ, cut the spinal marrow across, and thus, in a few minutes, two persons died by the same kind of accident!

Our author relates a series of cases illustrating the comparative fatality and danger of wounds and contusions, according to their magnitude and the part of the spine injured. Thus Duverney relates a case, where a pistol ball fractured the second cervical vertebra, which was driven in on the spinal marrow. The man lived four days, most part of the time insensible, speechless, and paralytic. Morgagni, on the other hand, gives us a case where considerable injury must have been done to this organ, without death ensuing. A young man was wounded by a stiletto in the upper part of the neck, near the origin of the spinal marrow. He fell down on the spot, deprived of sense and motion, and in this state he was carried home. As he appeared to be very cold, the attendants imprudently applied a considerable degree of heat to the lower extremities without his feeling the applications. The consequence was, that deep and sloughing ulcers ensued. There was at first retention of urine, and also of fæces—but in a few days both came away involuntarily. About the eighteenth day he began to recover a little feeling in the left side of the body, which was slowly followed by the power of motion. In six months he was able to walk a few steps.

Two cases are related of injury about the fifth cervical vertebra. As it is proper that young practitioners should be acquainted with the phenomena presented, and results to be expected from injuries at different distances from the summit of the spinal column, we shall be rather more particular than may, at first sight, appear necessary.

*Case.* On the 27th of September, 1819, a stone-mason in Paris fell from a ladder, and in his descent the back part of his neck was struck violently against one of the steps. He was carried to the Hotel Dieu, with paralysis of the upper and lower extremities, insensibility and coldness of the skin, laborious respiration, slow pulse, sighing, feebleness of voice, difficulty of answering questions, respiration carried on entirely by the diaphragm. The patient was threatened with suffocation. He died on the 29th, and was most part of the time sensible. On dissection, the fifth cervical vertebra was found fractured, and a portion of it driven against the spinal marrow, which was compressed to one half its ordinary volume.*

The following case is interesting, particularly as affording a rare example of dislocation, without fracture of the spine. It was, however, in the cervical portion, where we believe it can alone take place.

*Case.* On the 10th of December, 1821, Peter Jalet, having carried a sack of flour on his head and shoulders up a flight of steps, threw the sack down in the granary; but in this last act, felt something crack in the lower part of his neck, accompanied by a sudden and acute pain. He instantly fell down in a state of paralysis, and in this condition was carried to the Hotel Dieu. Here the paralysis of the inferior extremities was found to be complete, that of the superior incomplete. There was a slight degree of sensibility of the skin. He said he felt a sense of formication in his limbs, and cutting pain in his shoulders. There was paralysis of the rectum and bladder. The respiration was entirely carried on by means of the diaphragm, the thoracic parietes being quite motionless—pulse frequent and strong—skin hot—mental faculties unaffected. Bled from the arm, and 30 leeches applied to the back of the neck. 11th. Same state. Bled again both locally and generally. In the night the dyspnœa became very urgent, with great fever and delirium. He died next morning of suffocation.

*Dissection.* In the head nothing particular was observable. In the spine was found rupture of the intervertebral ligament, which unites the sixth and seventh cervical vertebræ, together with some other of the minor ligaments. The body of the sixth vertebra was pushed more forward than that of the seventh—and in this place the substance of the spinal marrow was violently contused and disorganized, but its envelopes not ruptured. The lungs were gorged with black blood, and very heavy. The lining membrane of the bladder was red and inflamed. The other viscera sound.

---

* Sometimes, where there is fracture of the spine, the medulla spinalis will be fatally injured, without the envelopes appearing at all affected. Dr. Gordon has related a case of this kind in the Edinburgh Medical and Surgical Journal, for October 1817. The case was that of a chimney-sweeper, who fell down a chimney, and fractured the seventh cervical vertebra. The theca vertebralis and arachnoid opposite the fracture appeared quite natural —the pia mater exhibited a bluish tint. But the organization of the cord itself opposite the fracture was completely destroyed for the space of half an inch, being reduced to a pultaceous mass not much thicker than cream.

In this, as in the preceding case, death evidently was occasioned by the lesion of the respiratory function. When the accident to the spinal marrow is below the origins of the phrenic nerves, M. Dupuytren thinks that inflammation of the medulla is propagated upwards, and having reached the origins of the phrenic nerves, asphyxia ensues.

In the following case the spinal marrow was almost obliterated by a fracture of the spine at the eleventh dorsal vertebra, and yet life went on for several days. The patient was a mason, who fell from a stage, and struck his back against some scaffolding in his descent. He was carried to the Hotel Dieu in a state of paraplegia, on the 25th of June, 1821. He was bled pretty freely. He presented the following symptoms:—lies on his back—features altered, and face covered with sweat—respiration free—pulse natural—no inclination to pass water or fæces, although the bladder was distended—loss of feeling and motion in the inferior extremities, except a sense of formication in the feet. 26th. Vomitings—pain in the epigastrium—pulse frequent. 27th. Vomitings have ceased, but in other respects he is the same. 28th. Ditto. 29th. The vomiting has returned, with increase of the epigastric pain—blood in the urine when drawn off. 30th. Much vomiting—swelling and tension of the abdomen. 1st July. Died.

*Dissection.* The body of the eleventh dorsal vertebra was fractured, and the vertebral canal almost obliterated at this part by a fragment of bone. The spinal marrow was, of course, greatly compressed, but its envelopes were not torn, though much ecchymosed. There was some phlogosis in the mucous membrane of the stomach and bowels; while the coats of the bladder were thickened and highly inflamed.*

---

* In the 20th volume of the Medical and Physical Journal is a case of injury of the spine related by Mr. J. Dorr of New York. A man received a blow across the back by the fall of a tree, which partially dislocated his spine, between the first and second lumbar vertebræ, producing a considerable tumour, and obtuse angle of the spine, with an entire abolition of sensation and motion in the lower extremities. Our author says he reduced the bones to their places. Next day he found the bladder violently distended, and also retention of fæces. The water was obliged to be drawn off for eight days, when an involuntary discharge took place. The rectum continued paralytic ever afterwards. "For about three years past his urine has been discharged in the following ways, viz. about one fourth passes through a sinus, nearly in a lateral direction from the umbilicus on the right side—one-third through the urethra—and the remainder through the most depending part of the scrotum, through several openings." It was curious that the testicles were entirely obliterated. The lower extremities were greatly emaciated, but the upper part of the body was plump and firm. His digestive and intellectual functions were untouched.

It is curious that the interruption of nervous influence should give a tendency to inflammation in a part. It is certain that concussions, contusions, and fractures of the spine, almost invariably produce inflammation of the lining membrane of the bladder. The urinary secretion is also greatly deranged in these cases. Dupuytren has remarked, that in paraplegia, of all other diseases, the catheter, when left in the bladder, is soonest covered with a saline incrustation.*

The few cases which we have extracted from a great number collected by our industrious author, present the principal phenomena resulting from injuries by violence to the spine. It is needless to say that, in such cases, our prognosis must almost always be of the sombre cast; for few indeed are the recoveries after serious lesion of the spinal marrow; and, consequently, such rare exceptions are not to induce us to betray ourselves by expressing much hope of recovery in any case. At the same time we may be deceived in our estimate of the extent of lesion in the spinal marrow, and therefore, while we warn the friends of the great danger of the case, we should take the precaution of giving the patient every chance of recovery. The removal of pressure, where it exists, and the prevention or repression of inflammation, which is pretty certain to ensue, are

---

* In the 25th volume of the Medical and Physical Journal Mr Harrold has related an interesting case of fracture of the vertebræ of the spine. A man was at work in a chalk-pit when the vault fell in and buried him several feet under chalk and flints. When seen, there was evidently fracture of the last dorsal and first lumbar vertebræ, the back there forming a considerable angle, with complete paraplegia. He was placed in a fracture bed invented by Mr. Harrold. It was necessary to draw off his water daily, and after some time the urine was found to contain a good deal of offensive purulent matter. In a few weeks the power of voiding his water returned. In spite of all their care the integuments over the sacrum sloughed. In about six months the following report was made by Mr. Harrold. "His back is as straight and flexible, and apparently as strong as ever—for he can sit perfectly erect in a chair, and can stoop sufficiently, as he sits, to rub his feet on the ground." The spinous process of the fractured vertebra is still more prominent than the others. He retains and passes his urine as he pleases. "He has a stool every two or three days, and begins to be conscious of passing it. No feeling or power in the lower extremities—appetite and bodily health good." In a subsequent paper in the succeeding volume of the same journal, Mr. Harrold informs the profession that the sacrum proved to be diseased where the slough had been situated; the discharge became profuse, and he died hectic one year subsequent to the fracture. On dissection, it was found that the bodies of three or four vertebræ had been crushed and fractured, more or less, but all the fractures were firmly united by bone. A splinter had been driven in upon the medulla spinalis, its sheath burst open, and the medulla itself nearly destroyed.

almost the only means which surgery and medicine have in their power. We need not descant on these.

§ 4. *Slow Compression.*—There are numerous circumstances, independent of external violence, which cause compression of the spinal marrow in a more or less slow manner. Thus sanguineous effusions into the theca vertebralis will produce compression very quickly, while curvatures and other affections of the osseous fabric will be very slow in operation. These last are the more common instances of slow compression. Exostosis of the vertebræ sometimes infringe on the vertebral canal, and strangle the spinal marrow by degrees. We shall here introduce a curious case.

*Case.* Nicolas Petipas, 60 years of age, a gilder, and ricketty, had never been able to walk without crutches, on account of an immense lateral curvature of the spine. He entered La Pitie in 1820, where he got cold in a damp ward. He began to complain of deep-seated and constant pain in the back of the head and along the cervical vertebræ. This pain gradually increased, and became very acute, when he attempted to eat or drink. The motions of the head became gradually impeded, and he felt a crackling kind of noise in his neck. This state continued six months; and he was then sent to the Bicetre, where the same symptoms continued. In the beginning of 1821, it was perceived that there was a prominent tumour in the nape of the neck, and that his head drooped forward. It was at this time that our author examined the patient. The whole neck was numbed, as were also the integuments of the upper part of the chest, the upper and lower extremities, the trunk as far as the umbilicus. In these parts was felt a stinging or tingling sensation, which could not be quieted. It was curious that there was a zone of about six inches in breadth quite round the chest, where the skin preserved its natural sensibility, and was free from the tingling sensation abovementioned. There was no affection of the digestive or urinary organs. In September 1822, he began to lose the power of his arms, which was soon followed by a similar affection of the lower limbs. The breathing was impeded, but not to a great extent. Gradually paralysis complete of the abovementioned parts became established, while the sensibility continued in the zone of integuments of the chest. Gangrenous eschars now formed on the sacrum, trochanters, &c. and this poor wretch's existence was terminated on the 31st of December, 1822.

*Dissection.* The substance of the brain was firm, and without any notable injection of the vessels. There was about half an ounce of turbid serum in the right lateral ventricle. No other alteration in the head. *Spine.*—The second cervical vertebra was enlarged and diseased, and caused the prominence in the nape of the neck. It encroached on the vertebral canal internally. The two first lumbar vertebræ were also diseased—their intervertebral cartilages destroyed in part, and about an ounce of serous effusion in the arachnoid spinal cavity at this place. Opposite to the diseased vertebræ, both cervical and lumbar, the dura mater was thickened and diseased, and adherent to the arachnoid. The pia mater, at this point of the spinal marrow, was red and adherent to the other envelopes. Anteriorly and below the corpora olivaria the pia mater was changed in colour, and its vessels greatly gorged with black blood. Opposite the second cervical vertebra there was a depression or indentation of the spinal marrow, about five lines in extent, and a line and a half in depth. The substance of the spinal marrow, in this place, was softened and pulpy. Abreast of the *sixth* cervical vertebra another softened portion of spinal marrow was seen, extending to the second dorsal vertebra—and a third diseased portion, from the seventh dorsal vertebra down to the cauda equina, which was reduced to a kind of pultaceous mass. The nerves issuing from these diseased portions of spinal marrow did not exhibit any appreciable alteration in their texture. The thoracic and abdominal organs were sound.

The reader will not fail to perceive that this dissection elucidates the paralysis and formication, which corresponded exactly with the diseased portions of spinal marrow. The zone on the chest of undiminished sensibility is also accounted for by the corresponding portion of unaltered medulla spinalis.

Our author remarks that in curvatures of the spine, anterior or posterior, in ricketty people, there is no danger of compression of the spinal marrow; as the curve is always so gentle as not to incommode that organ. In lateral curvatures, however, the case is altered, as there the nerves issuing from the spine are compressed on the concave arcs of the curvatures. Another cause of slow compression of the spinal marrow is enlargement of the intervertebral cartilages, which is sometimes considerable, and is not unfrequently observed in those who die of caries of the spine. The immense enlargement of the spinal veins in old people is another cause, in our author's opinion, of compression of the spinal medulla. So also may be enumerated, in the catalogue of causes, effusions of every kind in the cavity of the spinal arachnoid—the presence of hydatids, exterior or interior to the envelopes—tubercles.

In respect to the symptoms attending these states, we must expect that they will be less prominent in proportion as the compression is slow in its progress, and extended over a great space of the spinal marrow. The principal symptoms will, of course, consist of diminution, more or less considerable—in short, paralysis more or less complete, of feeling or muscular power, or of both at the same time, together with abolition of the functions of the rectum and bladder. In the beginning of the disease, there is often a numbness, then convulsive twitchings of the muscles, and finally paralysis.

In respect to the treatment of compression, we need not say any thing. The disease is too often fatal, when the cause is a slow disorganization, whether in the envelopes, or bony fabric of the spine. When the compression is produced by fracture and depression of the vertebræ, our author thinks that trephining the spine may be a proper measure, since death will be inevitable, unless the compressing power is removed. Here our author gives a very full account of Mr. Tyrrell's operation, as communicated to him by his friend Dr. Georgi, of Bologna, who assisted at the operation. As the English public are not yet acquainted with the case in detail, we shall here introduce the particulars of the case.

"On the 17th of October, 1822, a man, about 30 years of age, was brought into St. Thomas's Hospital, who had received a fall on his back, while carrying a heavy burthen. He had hemiplegia, and a fracture, with depression, of the tenth dorsal vertebra, was easily recognized. Sir Astley Cooper, Mr. Travers, Mr. Green, and Mr. Tyrrell, consulted together, and determined on an operation, which was performed the same day in presence of a great concourse of spectators. Being placed on his face with the spine a little bent forwards, an incision, four inches in length, was made directly along the spinous processes of the four last dorsal vertebræ, and the muscles dissected back on both sides, so as to lay bare the arches of the ninth and tenth vertebræ, on the latter of which the crown of a trephine was applied, but it would not work there. A chain saw was then employed, which removed the spinous process of the tenth vertebra at its base, so that the finger could be introduced under the portions of arch on both sides, which were then easily sawed off. The same was then done on the ninth vertebra, which had been fractured and depressed. About three inches of the spinal marrow and its envelopes were thus laid bare, and it was seen to pulsate, or swell and retract very distinctly. The wound was dressed simply. In a few hours after the operation the patient had some return of feeling in the lower extremities, which he had not had before since the accident. This return of sensibility, however, was only temporary. A few days after the operation he passed voluntarily both stools and urine. The latter was bloody. Paralysis still continued. Finally, the patient sank twelve days after the operation, with all the symptoms of peritonitis and enteritis in a severe degree. These last were amply proved on dissection, which also shewed the mucous membrane of the bladder very red, and the coats thickened. The dura mater covering the portion of spinal marrow denuded by the operation was black. As a preparation was to be made of the parts, no farther examination was made of the interior of the medulla spinalis." 224.

§ 5. *Commotion or Concussion of the Spinal Marrow.* This may be produced by falls or blows on the spinal column, or even on the hips or feet. The symptoms are nearly the same as those attending wounds or contusions of this organ, to wit, paralysis more or less complete of feeling and of motion; retention or involuntary discharge of urine and fæces; sometimes convulsions; and sometimes curious and anomalous symptoms, as we shall see in the detail of cases. Dr. Abercrombie, who has written a very interesting paper on diseases of the spinal marrow, in the Edinburgh Journal for January 1818, observes, that "paralysis of the lower extremities and *suppression* of urine are the symptoms that most frequently come under our observation." In this quotation, and indeed throughout the paper, Dr. A. has strangely used the terms *suppression* and *retention* of urine indiscriminately and synonymously, though they signify quite different states, and the *former* is not a concomitant, or very rarely so, of affections of the spinal marrow. He justly remarks, that concussion of the spine may prove speedily fatal, without leaving any appreciable organic change in the part after death. Boyer, Frank, and others, relate cases of this kind. Dr. Ollivier observes, that the effects of the concussion will be in proportion to the violence of the injury. Sometimes we find, after death, sanguineous effusions between the theca and the spinal marrow—sometimes the pia mater is burst in one or more points; as is the case occasionally even with the dura mater and arachnoid covering, the medulla then forming a hernia or protrusion at that part. Sanguineous effusions are also found in the cavity of the arachnoid membrane, when the concussion causes rupture of some of the small vessels. When there is appreciable alteration in the spinal marrow itself, it usually consists of a softening of the medullary substance, the product, our author believes, of inflammation. In these cases we find the vessels of the pia mater injected with blood.

If none of these organic lesions have taken place, the patient has a much better chance of life. The symptoms gradually lessen in force, and the patient recovers. We shall now proceed to an elucidation of this disease, which is doubtless much more frequent than is believed, by a detail of cases more or less copious.

*Case* 1. Morgagni, in his 54th epistle, relates the case of a man who was struck by a branch of a tree on the three last lumbar vertebræ. He was carried to the Santa Maria Hospital, and died in four hours. There was no fracture of the vertebræ; but there was an effusion of blood within the vertebral canal, though the medulla spinalis appeared at this part quite sound. All the viscera were sound, and the arteries were observed to be full of coagulated blood.

The following extremely curious case was carefully examined by our author's father, and, also, by M. Billard, in the Hotel Dieu of Angers.

*Case* 2. A woman, 49 years of age, who had previously enjoyed good health, began, in the year 1820, to feel strange pains through her body, originating in the left side of the chest, and caused, as she thought, by a small scirrhous tumour which was developed in the left mamma, but which was not painful on pressure. These pains increased in intensity, and deprived her of all enjoyment of life. She tried intoxication, but that rather aggravated than alleviated the evil. Still the tumour in the mamma remained stationary, and never became sensible to pressure. The unhappy woman was for ever recounting the miseries she suffered; and, at last, tired of existence, and wishing to deliver herself from the tortures she endured, she threw herself, on the 5th November, 1822, from a window in the fourth story of the house where she lodged. She was carried to the hospital, where our author saw her immediately, with the following symptoms:—Stupor, pallor of the face, great dyspnœa, pulse small and slow, skin cold, paralysis and insensibility of the trunk and lower extremities. There were various injuries of different parts of the body, which we shall pass over. She died on the evening of the 8th November.

*Dissection.* In the head, the meninges were found thickened, but very little water in the ventricles. The body of the 10th dorsal vertebra was fractured transversely, but without the slightest displacement of the fractured portions. In the vertebral canal, opposite the fracture, there was an extravasation of fluid blood on the exterior surface of the dura mater. The medulla spinalis appeared of its natural colour and consistence.

In the thorax, the small scirrhous tumour was examined, but presented no trace of inflammation or disease around it. There were various fractures of the ribs; but the cause of the patient's long-continued sufferings was now brought into view. In the left side of the chest, and just above the arch of the aorta, was found a tumour of a pyriform shape, the size of a hen's egg, covered by, and adherent to, the pleura on one side, and on the other, it was in contact with one of the dorsal vertebræ. From the upper part of the tumour arose a white cord, the size of a goose-quill, which was traced into the foramen between the first and second dorsal vertebræ. On examining the spinal marrow, at this part, the filaments of the anterior and posterior roots of the first dorsal nerve were distinctly seen directing their course, as usual, to the foramen, for the formation of the first dorsal. This nerve, after issuing from the spine, gave off, as usual, a posterior branch. The anterior branch (after communicating with the great sympathetic, and giving rise to an ascending twig that passed on to unite with the seventh cervical) suddenly enlarged in volume, and proceeding about an inch from the point where it gave off the ascending twig, it terminated in the summit of the tumour. In this course, its colour was not altered—its neurilema was thickened, and it lost itself in, and became blended with, the tumour in question. This tumour was white and elastic, presenting some cartilaginous spots on its posterior surface. On slitting open the nerve which served as a peduncle, the neurilema of the nerve was found to expand itself and form a sheath, as it were, for the tumour. This last, when slit open, presented a pulpy surface, of a whitish colour, and appearing to be formed of concentric fibres, without a trace of vessel of any kind in its composition.

Our author remarks, that there are many cases recorded where scirrhous tumours of this kind were found developed in the substance of nerves; but none, he believes, where the nerve terminated in a tumour. Generally, the scirrhous tissue of these tumours is strewed with vesicles or small cysts containing a fluid. Here then was, no doubt, the cause of the patient's sufferings, which could not be accounted for during life, and which were, in all probability, considered as hypochondriacal. We think it highly probable that, in many of those cases called monomania, where patients complain of dreadful sufferings that are regarded as ideal or the offspring of insanity, there would be found, if the bodies were minutely examined after death, some *physical cause* for their complaints. This woman was reduced before the act of suicide, to a state of great emaciation. This, we should suppose, was from her sufferings and mental misery, rather than from any mechanical effect which the nervous tumour could produce. The great difficulty lies in explaining how this tumour gave origin to such dreadful pains as ultimately destroyed the poor woman's reason. Was it from the stretching of the neurilema and nervous fibres themselves during the development of the tumour? We should be inclined to this conclusion.*

*Case* 3. A man, 46 years of age, was brought into the Hotel Dieu of Angers, on the 17th October, 1817, who had had a fall on his back, where he complained of great pain. The lower extremities were paralyzed, with retention of urine and fæces. The pulse was frequent. This state continued till the 31st, when delirium took place, accompanied by swelling and tension of the abdomen. He died on the 3d November, in a state of great difficulty of the breathing.

*Dissection* Nothing particular in the head. On opening the spinal canal, the coverings of the spinal marrow were found rent in some places, and the substance of the medulla forming herniæ at these points. There was, also, an extravasation of a sero-sanguineous kind between the

---

* It is curious how the presence of a morbid growth in an internal part will often cause great functional disturbance, even where there would not appear to be much mechanical inconvenience incurred. We lately assisted our ingenious friend Mr. Bampfield, in opening the body of a female who had come into the Covent Garden Workhouse to die, in the last stage of emaciation, attended with incessant dry cough, excessive dyspnœa, and almost complete inability to swallow. She died of inanition and difficulty of breathing. On examination, there was no disease in the trachea or œsophagus. The right lung was diseased; and just at the bifurcation of the trachea, and anterior to it, but apparently not pressing on it, there was developed a solid, irregular, semi-cartilaginous tumour, the size of a small hen's egg, apparently a diseased cluster of bronchial glands. The trachea and œsophagus were not altered in calibre or appearance at this place.—*Rev.*

membranes and medulla. The lungs were gorged with blood, but sound. There was some peritoneal inflammation and effusion. The coats of the bladder were thickened and inflamed.

Concussion of the spine, though often fatal, is not always so.

*Case* 4. Antony Majourel, 45 years of age, fell from a height of 36 feet, on uneven ground, by which fall he was rendered senseless. In an hour and a half after the accident, Dr. Combaldieu saw him. His intellectual faculties were disturbed, his pulse small and concentrated—feeling and motion of the lower extremities completely abolished. Two contusions were observable—one on the right arm—the other on the dorsal region about the 10th and 11th vertebræ. He was five times bled in the course of the day—the water drawn off by catheter. Next day the patient had nauseæ—the tongue covered with a mucous crust. An emetic was administered in a lavement, and operated. In this state he continued for the five following days. On the 11th day he began to feel some tingling sensations in the left leg, and after some smart frictions of the limb with a brush, he evinced some slight muscular power in the same. At this time, the power of voiding the urine returned. By the 20th day, the power of the left lower extremity was entirely restored—the right was still paralyzed. In a month both members were restored to feeling and motion.

Camper relates the case of a soldier who, in a paroxysm of phrenzy, leaped out of the window of a second floor, falling first on his feet, and then prostrate on his back. He was taken up paralytic of the lower extremities, with involuntary discharge of urine. He required a whole year to recover from these. He afterwards died of a putrid fever, and Camper had an opportunity of examining the spine. One of the lumbar vertebræ had been fractured transversely in its body and united completely. Camper preserved the specimen.

*Case* 5. Charles Cagniaux, 22 years of age, fell from a fourth floor on his back and hips. He was carried to the St. Louis Hospital on the 11th June, 1821. There was observed a considerable contusion in the lumbar region and back part of the pelvis;—suspicions were entertained that there was fracture of some of the vertebræ;—complete paraplegia. Bled copiously from the arm, and numerous leeches applied on the site of the contusions. He continued in this state for several days. His water was drawn off twice a day—evacuation of fæces involuntary. Bleeding and the application of the moxa were repeatedly employed. Gradually the power of expelling the water returned, and became involuntary—the alvine action was still involuntary. At present, 4th May, 1823, the man enjoys good health; but he has not completely recovered the motion of the lower extremities. He walks by the aid of a stick, and is very liable to fall down. He has no feeling in the skin of his feet, as far as the ancle-joint; the motions of which are almost entirely lost. The sensibility is also lost in the *posterior* surfaces of the legs, thighs, and hips, while it is preserved entire on the opposite sides. The evacuation of urine and fæces is still involuntary, in spite of every means that have been tried to remove so dreadful an evil. No deformity can be detected in the spinal column. In the above case, the sciatic nerves appear to have suffered, while the crural nerves have preserved their integrity.

*Case* 6. The remarkable case related by Mr. Charles Bell, in Part II. of his Quarterly Reports, was evidently a case of concussion of the spine, ending in suppurative effusion. The man was a waggoner, who was thrown off the shaft of his cart by a sudden jerk, pitching on his back and shoulders. He was carried to the Middlesex Hospital, where he lay for seven or eight days without complaining of any thing, except stiffness of the back of the neck, being able to move all his limbs with freedom. On the 8th day he was seized with general convulsions and trismus, which last changed in a few hours, to a singular convulsive motion of the jaw, and this again to maniacal delirium. He now sunk into a typhoid fever, and in four days was found to be paralytic of his lower extremities. He died about a week after this period. *On dissection*, a quantity of purulent matter was found within the spinal canal, especially at the lower part of it. It appeared to have been formed about the last cervical, or first dorsal vertebra, and there the intervertebral cartilages had been eroded, so that the pus had escaped outwards among the muscles.

*Case* 7. In the 17th volume of the Edinburgh Medical and Surgical Journal, Dr. Arthure, of Dublin, has detailed a very curious case of concussion of the spine in a lady, 52 years of age, who, on the 1st December, 1818, fell, by accident, off a chair on which she was sitting, and received a contusion on the os coccygis. The pain soon subsided by quietude, and in a few days, went entirely off; but, on the 20th of the same month, while sitting at needlework, she suddenly cried out that she was losing her senses—had got an excruciating pain in the forehead and temples—and feared that it would terminate in apoplexy. She was bled by the family apothecary, and was soon afterwards seen by Dr. A. who found her in a very alarming state of exhaustion. The pulse was scarcely perceptible—extremities cold—constant vomiting. On recruiting a little, 20 leeches were applied to the head, as the pain there continued as violent as ever. Dr. Crampton, on being called in, concluded with Dr. A. that the complaint was ascribable to the fall, and that the present symptoms were referrible to an affection of the brain, from sympathy with the spinal marrow. Blisters were applied to the calves of the legs, and a stimulating cathartic enema thrown up. Cold was, also, applied to the head. By these means she became convalescent; but, on the 4th January, she was again seized with excruciating pain in her head, "along with irritating sensations about the os coccygis, bearing-down pains in the uterine region, and spasms in the calves of the legs. She, also, felt an indescribable sensation of a painful nature, ascending along the dorsal vertebræ, and terminating about the foramen magnum of the occipital bone." In this attack, anodyne antispasmodic medicines succeeded, in conjunction with leeches to the sacrum, in removing the complaint.

*Case* 8. Dr. Abercrombie mentions the case of a man who had been employed in blowing up the rock near Edinburgh, and who was struck by a large piece of stone on the spine, about the lower dorsal and upper lumbar vertebræ. He instantly fell, completely deprived of the power of the lower extremities. When seen, there was such extensive swelling that it could not be ascertained whether or not there was fracture of any of the vertebræ. He was confined to bed for several weeks paralytic in the inferior members, and with considerable difficulty in passing his water; but he gradually recovered.

*Case* 9. In Hufeland's Journal, is related the case of a man who fell from the top of a loaded cart and pitched on his neck and shoulders. When recovered from the shock, it was found that he was paralytic below the neck—he could move no part but his head. He had retention of urine and obstruction of the bowels. After eight or ten days he became affected with swellings of the limbs, and a sense of prickling, followed by severe pain, but without any power of motion. He very gradually and slowly recovered.—*Abercrombie.*

*Case* 10. Dr. Abecrombie observes (loco citato) that concussion of the spinal marrow may produce permanent paralysis either immediately, or after a considerable interval of time. In illustration of this position, Dr. A. relates the case of a man, 43 years of age, who, about nine years previously, had fallen from the branch of a tree, and lighted on the sacrum. He was carried home deprived of the power of his lower extremities, but in ten or twelve days, recovered so far as to be able to follow his avocations. From this time, he was affected with a peculiar sense of numbness in the upper part of the left foot. After continuing in this state for four years, the numbness suddenly extended up along the same leg and thigh, and was speedily followed by paralysis of these parts. Paralysis of the other leg and thigh soon followed, and he was confined to bed for two years with complete hemiplegia. Afterwards, he recovered partially the use of the lower extremities, so as to be able to crawl about. His spine was free from distortion, but he complained of deep-seated pain, on pressure, about the last dorsal vertebra and top of the sacrum. At this place two caustic issues were inserted, and considerable improvement followed. In such cases, Dr. Abercrombie thinks the nature of the disease is probably that of chronic inflammation of the spinal cord or its envelopes.

Before we close the subject of concussion, we may say a word or two respecting the treatment. In this, as in concussion of the brain, we should not fly to the lancet the moment a man has met with an accident, merely because he has received one. We should allow the first effects of the shock to recede, especially if there be faultering of the pulse, and paleness of the skin. When these are over, then we may safely deplete and counter-irritate, not only to prevent, but to control re-action, which sooner or later must come on.

§ 6. *Effusions into the Spinal Canal.*—These are of different kinds, and in different situations. Thus, they may be of a liquid or gazeous nature;—and they may be external or internal of the dura mater and arachnoid coverings. Of the liquid effusions or extravasations, there have been observed several kinds, as serous, purulent, sanguinolent, &c.

A. *Sanguineous Effusion—Hœmatorachis.*—In accidents from external violence, we often find blood extravasated within the spinal canal. When this effusion is within the cavity of the spinal arachnoid, it often originates in the head, and descends along the spine. Of the latter kind is the following case.

*Case* 1. Dessieres, 26 years of age, received, on the 30th November, 1822, a foil thrust on the left temple, by which he was felled on the spot. Conveyed to the Hotel Dieu of Anvers, he appeared in a profound sleep, breathing slowly and laboriously, with froth issuing from his mouth, and slight convulsive twitchings agitating his limbs. He was bled from the arm, without any sensible effect. He died three hours after the accident.

*Dissection.* The meningeal artery was torn, (there being a perforation of the bone) and considerable effusion of blood on the surface of the brain, and also along the base of the skull. The meningeal cavity of the spine was filled with fluid blood.

The point of the foil had penetrated an inch into the substance of the brain.

*Case* 2. Bonetus relates a case of sanguineous effusion in the spine, from spontaneous rupture of vessels at the base of the brain.

Ann Walterin, 70 years of age, had been in the habit of frequently losing blood by venesection. She went out one morning to gather wood, but did not return. She was found dead, with a very slight wound on the left temple. Bonetus examined the head, and pronounced that the wound was merely from falling down, death being produced by an internal cause. A great extravasation of blood was found between the pia mater and brain at its base, and thence extending down through, and filling, the spinal canal. The blood issued from small arterial branches arising from the carotids and vertebrals at the basis of the brain.

*Case* 3. Mr. Chevalier relates, in the third volume of the Medico-Chirurgical Transactions, an interesting "case of spontaneous extravasation of blood within the theca vertebralis, which soon terminated fatally," occurring in a girl of 14, accompanied by pain in the back and head, sickness on sitting up, and quick pulse. In about a fortnight, she complained of a sudden and violent increase of pain, was immediately seized with convulsions, and died. On dissection, the brain and viscera were found healthy, and the spine was free from any external appearance of disease. But, on opening the spinal canal, it was found filled with blood, imperfectly coagulated—"it is, therefore, probable, that the vessel first gave way at the commencement of the illness, and again, to a much greater extent, at the time the convulsions came on." Mr. Chevalier mentions another case somewhat analogous, occurring in a child only a year old. These cases he considers as a sort of partial spinal apoplexy, which should be treated by early bleeding, in order to prevent the increase of the extravasation.

*Case* 4. In Mr. Howship's "Observations in Surgery," there is related the case of a boy, 14 years of age, who received a violent jerk of his neck by a cord which was thrown over his head while in a swing. He felt no bad effects at the time, but after a time, he was observed to be weak and inactive, becoming gradually more so, with stiffness of the neck, and difficulty in moving his head. In nine months after the accident, the weakness in the lower extremities amounted to paralysis, which was speedily followed by the same in both arms, with retention of urine and obstinacy of bowels. In a short time after this, he was seized with very violent pain in the spine. His breathing became quick and laborious, and he soon died. On dissection, a quantity of extravasated blood was found in the spinal canal, lying between the bone and the theca vertebralis, partly coagulated and partly fluid, appearing to have come from the upper part of the canal, about the second or third cervical vertebra. We shall conclude the subject of Hæmatorachis with the substance of a remarkable case recorded by M. Gaultier de Claubry in the Journal Général de Medecine, for 1808.

*Case* 5. M. Durand, 61 years of age, who had always enjoyed good health, retired to his country-seat from the storms of the Revolution, where he spent his time in agricultural pursuits. In August 1808, he came up to Paris, and the day after his arrival found himself rather unwell, when Dr. De Claubry visited him. He complained of pain along the spine, which he said he had felt, in a degree, for some time past, accompanied by a sense of weight and numbness which extended to the thighs, legs, and even to the feet. These last symptoms he attributed principally to the fatigue of travelling up to town in a voiture, where he was incommoded by too many passengers. The vascular system was tranquil, the skin of its natural temperature, the pulse was rather full. All the other functions in a state of integrity. He was advised rest, and the warm bath. He was visited at 10 o'clock in the morning. At 11, Dr. De Claubry was summoned, and informed that the patient could not move his limbs, and that both urine and fæces came away involuntarily. In this state our author found him. The pulse had lost some of its force, the breathing was not quite easy, the countenance was altered. While answering some questions put to him by his physician he expired.

*Dissection.* There was some water in the lateral ventricles of the brain, and blood flowed from the vertebral canal into the basis cranii, when the spinal marrow was cut across at the foramen magnum. When the spinal marrow was laid open, this fluid flowed out still more abundantly. When the envelope of the spinal marrow was slit open, this part was disorganized "*comme une bouillie d'un rouge sang de bœuf,*" an expression which we cannot readily render into English. This appearance presented itself from the sacrum to the second dorsal vertebra, where something like spinal marrow was seen. It was not till he got up as high as the 7th cervical vertebra, that the medulla spinalis appeared sound in substance, but of a deep red colour.

In attempting to slit the spinal marrow longitudinally, it fell to pieces under the scalpel, and it was only at its upper extremity, near the head, that it preserved its natural consistence. The interior of the organ participated in the same hue as the exterior, gradually diminishing in intensity as it approached the brain. There was sanguineous effusion between the membranes and the spinal marrow itself, throughout the greater portion of its extent.

B. *Serous Effusion.*—In opening the spinal canal there is very frequently found some serous effusion—indeed, more frequently and in greater proportion, (relatively to the extent of surface) than in other cavities of the body lined by serous membranes—whether that effusion percolates down from the brain, or is exhaled from the serous envelopes of the spinal marrow. This effusion is sometimes yellow and limpid—sometimes whitish and turbid—not unfrequently tinged with blood. Our author has remarked that, in general, the quantity of effusion was in proportion to the turgescence of the venous system of the medulla and its envelopes. The lentor and difficulty of the venous circulation in this region, account sufficiently for this augmented exhalation, and explain why we find more of it here, after death, than in other serous cavities. These effusions in the spine have been frequently observed in opening the bodies of old people who have died of apoplexy—and, also, of certain cerebral irritations. This serous effusion is sometimes accompanied by convulsions. Bonetus relates the case of a person who died phthisical, after having been, for twelve years, harrassed with spasms and convulsions of the trunk and members, which prevented him from being at rest for one hour at a time, night or day. The paroxysms, which were repeated at very short intervals, were succeeded by a languor and loss of voluntary power, amounting nearly to paralysis—then again the convulsive agitations. On opening the body, there was found an abundance of limpid serum in the anfractuosities of the brain and in the vertebral canal. The spinal marrow was notably decreased in volume, an effect attributed by Bonetus to the pressure so long-continued of the serous effusion. The same author, indeed, reports several cases analogous to the above, but not so remarkable in kind.

In cases of gibbosity of the spine, we not unfrequently find a good deal of serous effusion in the spinal arachnoid cavity. It is generally limpid, unless caries of the vertebræ has determined phlogosis of the membranes, in which case it is turbid. Concussions of the spine may produce serous effusions, but more frequently sanguineous. Here our author quotes a remarkable case lately published in M. Magendie's Journal, by Dr. Rullier. We shall give the prominent heads of the case.

Mr. L. 44 years of age, (at three years of age he had shewn some curvature of the spine, in consequence of which the right shoulder became elevated) for the ten preceding years had experienced some embarrassment in the movements of the arms, and, subsequently, some pain and numbness in the curved portion of the spine. On the 21st January, 1815, he fell, by accident, prostrate on the ground, from which he was unable to raise himself, till assisted. From this time, his upper extremities became contracted, stiff, and contorted. The point of curvature, or prominence of the spine, now became very painful and gradually more and more so. The right shoulder continued to rise, and the head to sink between the shoulders. He lost all power of the upper extremities. Blisters, moxas, and local bleeding did no good. With the exception of the upper extremities, all other parts of the body were unimpaired. The patient walked about. Although motion was lost in the upper extremities, they preserved their sensibility. For some time previously to M. Rullier's attendance the patient had coughed, and expectorated with difficulty. He now became devoured by hectic fever—and he died on the 31st October, 1822.

*Dissection.* The brain was very firm, and apparently quite healthy—some water in the ventricles. When the spinal canal was laid open, the spinal marrow did not appear to have suffered any compression—it took the twists and turns of the spine itself. There was a considerable quantity of serous effusion in the spinal arachnoid cavity. In some parts of the pia matral covering of the medulla there was much injection of the vessels, arterial and venous. When the coverings were slit open, the spinal marrow appeared sound from the head to the origin of the fourth cervical nerves. Two thirds of the dorsal portion (counting from below) were also sound; but, between these two portions—that is, for the space of seven inches, or more, corresponding to the origins of 8 or 9 pair of nerves, the spinal marrow had undergone a remarkable alteration. It was reduced to a state of almost complete fluidity, (veritable liquide) flowing out when the membranes were punctured. At the same time, it was found that some little trace of organization was still discoverable on the anterior portion of the spinal marrow.

We believe there are no characteristic symptoms by which we can distinguish effusions on the spinal marrow from some other affections of that part. They cause paralysis and, sometimes, convulsions; but so, also, do several other morbid conditions of the spine. There is a species of paralysis, however, which Dr. Ollivier thinks may be recognized during life, as resulting from spinal effusion—namely, the ascending or descending paralysis—that is, a paralysis which progressively marches from above downwards, or *vice versa*. Of this disease we shall present an example, which happened at the Hospital, Necker.

*Case.* Adolphus Desurmont, aged 20 years, a blacksmith, entered the Necker Hospital on the 11th October, 1822, presenting the symptoms of gastro-intestinal fever. He had, four times in the course of the fever, a profuse nasal hæmorrhage, without crisis; and the disease was protracted till the 30th day, when the symptoms began gradually to abate, and in 30 days more the cure appeared to be complete, except some debility. One evening, while walking in the ward, he felt a remarkable numbness in his lower extremities, which soon bent under him, and he fell to the ground. On being put in bed, he vomited up a quantity of yellow bile. His skin was hot and his pulse frequent. No disturbance of the intellectual functions. He could sleep none that night. Next day nearly the same. The paraplegia was incomplete, and the skin was the seat of a constant sense of formication. The numbness extended no higher than the epigastrium. He felt a pretty acute pain along the spine, as high up as the middle of the dorsal region. The upper extremities weie entirely free from paralysis. He continued the same till the fourth day, when the pain in the back increased, and extended up to the nucha, the arms being weakened and somewhat benumbed. There was still heat of skin with quickness of pulse. On the fifth day, the numbness of the upper extremities was increased, as well as the dorsal pain. A gangrenous eschar, also, appeared on the sacrum. On the sixth day there was less numbness in the upper extremities, and the amelioration gradually went on, from this time, from above downwards, till the lower extremities entirely recovered sense and muscular power, and the patient, ultimately, was discharged from the hospital cured. Our author thinks, and with reason, that there was, in this case, some inflammation of the coverings of the spinal marrow, terminating, probably, in slight effusion, which was ultimately resorbed.

C. *Gazeous Exhalation into the Cavity of the Spinal Arachnoid; or Pneumorachitis.*—There are numerous cases on record of gazeous collections in different parts of the body, but none, that our author could find, where the arachnoid cavity of the spine was the seat of the pneumatosis. It is not uncommon, he remarks, to find the lumbar portion of the meningeal canal distended by a gazeous fluid, inodorous and untinged, which cannot be attributed to putrefaction, as it is seen in subjects recently dead, and not found in bodies that have been long in a state of putrescency. In a woman, 65 years of age, who died of chronic peritonitis, our author found the meningeal canal in the lumbar region very much distended with a gazeous fluid, which rushed out as soon as he had made a puncture in the dura mater with the scalpel. There was, also, some liquid effusion here. The woman had been dead only twenty-two hours, and no signs of putrefaction were present. In another woman, who died of chronic colitis, a frothy serous effusion was found in the lumbar portion of the meningeal cavity, the vessels of the meninges being exceedingly injected. In a little child, three years of age, who died of hydrocephalus acutus, the spinal dura mater was much distended, especially its lower two thirds, by a gazeous fluid, which escaped when the membrane was punctured. Six or seven instances of this kind are mentioned by our author. The subject is connected with an obscure but interesting question in pathology—the generation of gazeous fluids in various parts of the body, in a sudden and mysterious manner, very puzzling to the pathologist. That air and other gazeous fluids are generated in the stomach and intestines, in some other way than merely a chemical one, namely, by a species of secretion, has been often suspected; and, we think, with great reason. There is no other way of accounting for many tumours which suddenly spring up in other parts of the body also, of which we have seen some and heard of many others. Bonetus, (vol. II. p. 276) in his Sepulchretum, states, though not with sufficient minuteness, the case of a nobleman who had been a long time afflicted with a chronic disease; the nature of which he does not describe. He was seized with inflammation of the throat, and, at the same time, a tumour was developed on his back. He died; and, on dissection, when the tumour was opened, nothing but air escaped.

About six or seven months ago, we were consulted, by letter, respecting a lady, the wife of a medical gentleman residing in a distant part of the kingdom, who had several times become suddenly affected with a most tense and painful globular swelling in the region of the sigmoid flexure of the colon. In some of the attacks it arrived at its full size in six hours, lasted many weeks, gave dreadful torture, and suddenly disappeared, without any sensible evacuation from the system. In one of these attacks, a regular physician, a fellow of the Royal College of London, examined the tumour with great care. He pronounced it to be *a solid organized body;* but, when he was informed that it required but six hours for its growth, and that it had made sudden appearances and disappearances previously, he candidly confessed he knew not what was its nature or contents. During these attacks, there was no obstruction to the passage of the fæces, which were regularly discharged daily, so that there was no fair ground to suppose the tumour (which was excessively tense, even, and globular) consisted of fæcal accumulations. It was subsequently to the last attack that we were consulted; and we gave it as our opinion, that the tumour was of an airy or gazeous nature, situated either in one of the cells, between the coats, or in the cellular texture under the sigmoid flexure of the colon. We should be much obliged by any facts bearing on these sudden and mysterious productions, addressed to the care of the Editor of this Journal.

§ 7. *Spinal Arachnitis.*—It is exceedingly rare to find spinal, unaccompanied by cerebral arachnitis. The symptoms and post mortem appearances of arachnitis have been amply detailed by us in the 8th Number of this Journal, for March, 1822, to which we refer for particulars. In order not to break the chain of our analysis, however, we cannot entirely pass over this section of the work before us unnoticed.

Our author observes that, generally speaking, the arachnoid lining the dura mater of the spine, is found (where inflammation had existed during life) covered by a whitish, opake, or membraniform exudation, more or less adherent, and of greater or less extent. Even puriform secretions have been found on its surface. The membrane itself becomes opake, sometimes red, and often contains, between its laminæ, a fluid of various colours and consistency. It is rare, however, to find organic alterations of the spinal arachnoid, independent of structural alterations in the spinal marrow itself. Our author relates one example, which we shall here introduce.

*Case.* Jean Baptiste Hacquart, 13 years of age, of apparently good constitution, was brought to the Hopital des Enfans, on the 19th of March, 1823. No history of his previous symptoms could be obtained, except that, during the preceding summer, he had complained of severe head-aches. Eight days previously to his entering the hospital, he was suddenly seized with hemiplegia of the left side, without premonitory symptoms. The following were the phenomena now presented:—Face flushed—speech embarrassed—tongue white—loss of memory—cough—deglutition difficult—pulse feeble, slow—acute pain in the epigastric and hypochondriac regions on pressure. The patient's head was thrown backwards, with stiffness and pain along the whole of the spine, augmented by any attempts to raise him up in bed. The left, or paralyzed members were rigid, but not divested of their sensibility—involuntary discharge of urine. 21st. Bowels opened—cough less—deglutition more easy—pulse more developed—thirst—acute pain in the neck and along the spine. A repetition of leeches to the back. Same state till the 24th, when more muscular power was observed in the left arm, which was increased by the 25th. On the evening of this day, much head-ache. 26th. He could carry the left hand to the head, the lower extremity being still paralyzed—pulse very slow. This state continued till the 31st, when pains in the lower extremities became very severe. On the 6th April, the pains were so increased as to cause the patient to cry out with the agony. He had been taking the strychnine, which was now discontinued. On the 10th, nearly in the same condition—the trunk being now in a completely tetanic state of rigidity. The symptoms went on increasing, and death closed the scene on the 15th April.

*Dissection.* In the head was found a double-lobed tubercle, the size of two small nuts, pressing on the middle lobe of the *right* hemisphere of the brain—and in another part of the same lobe, there was another tubercle the size of a pullet's egg. In the parietes of the right lateral ventricle, was a third tubercle, and in several other places, tubercles of a small size. All these tubercles were encysted, and the purulent-looking matter which they contained was fluid, yellow, but of some consistence. These, by many people, would be called abscesses in the brain, but, we think, very improperly. The *parietes* of these cysts were formed of two distinct membranes, the exterior one of which presented numerous vascular ramifications running from the brain upon the cyst. Between the meninges was found a purulent exudation:—and in the spine, a similar exudation was discovered along its whole extent, between the arachnoid and pia mater. The latter membrane was red, injected, and thickened. The veins of the spine were unnaturally turgid; but the substance of the medulla spinalis was perfectly healthy in appearance.

Another case is briefly related of a child, about four years of age, who was carried to the Hopital des Enfans, on the 2d February, 1823. The symptoms were, great difficulty in swallowing—fixedness of the eyes—to which succeeded, in a short time, tetanic symptoms, as trismus, opisthotonos. Leeches, opium, frictions, and the warm bath, did no good, and the disease persisted till the 11th of the same month, when the child died. On dissection, the brain was seen to be much injected, and of a firm consistence. In the ventricles, the arachnoid was thickened and injected. *Spine.*—In the middle of the dorsal region, there was a reddish infiltration into the cellular tissue placed between the dura mater and the osseous canal. On slitting the membranes, their cavity was found filled with a serous fluid. The vessels spread over the surface of the pia mater were exceedingly injected in the middle of the dorsal region only, where the arachnoid membrane was covered with an albuminous concretion, for the space of about four inches. The substance of the spinal marrow was slightly injected at this place.

The following presents an example, also, of circumscribed spinal arachnitis.

Augustus Berard, 28 years of age, entered La Charite on the 4th of April, 1823, having come from the St. Louis Hospital, where he had been under treatment for a deep-seated pain in the lumbar region, which succeeded the lifting of a heavy burden in September, 1822. A blister had been applied to the part, and produced an ulceration that was very difficult to heal. On entering the Charite, he complained of general debility, depression of spirits—and he was much emaciated. On the 7th, the following symptoms were noted:—difficulty in articulating words, the intellectual faculties not appearing impaired—mouth drawn a little to one side—face rather flushed—the left arm partially paralyzed—involuntary discharge of urine and fæces. (Blisters, lavements, aperients.) 8th. Had several evacuations —mouth not so much drawn—face pale—inability to articulate. 9th. Insensibility to surrounding objects. Died next day.

*Dissection.* Vessels of the head gorged with blood—extravasation of concrete matter under the arachnoid covering the left hemisphere—serous effusion into the right lateral ventricle. In the spine, there was found a turbid whitish serous effusion (about 12 drachms) in the lumbar region—injection of the arachnoid for the space of an inch in this region—the vessels on the surface of the pia mater were injected—the spinal marrow itself apparently healthy.

### Observations.

Hippocrates, our author thinks, appears to have recognized spinal arachnitis under the denomination of *pleuritis dorsalis,* describing it as accompanied with acute pain in the back, difficult respiration, and terminating commonly by death, on the fifth or seventh day. If this period was passed, the patient recovered. We think Hippocrates knew little or nothing of the matter. His prognostic remark is decidedly erroneous, and, therefore, the probability is, that he meant some inflammation within the cavity of the chest, and not within the cavity of the spine. In by far the majority of cases, spinal arachnitis is connected with, or extended to, the arachnoid of the brain. Whether isolated or complicated, our author considers the two following symptoms as nearly pathognomonic of the disease: viz. —1st, General contraction of the muscles on the posterior part of the trunk, varying in intensity, from simple rigidity to the most violent *opisthotonic* contraction. "This was observed in cases where dissection shewed the arachnitis confined to the spinal meninges, and where those of the head were no way implicated." The second symptom is pain in some part of the back, varying also in intensity, and sometimes presenting remissions, or even intermissions. These symptoms, when present, may, he thinks, indicate, with certainty, spinal arachnitis —for in every instance where dissection took place after such symptoms, inflammation of the spinal arachnoid was found. There are other symptoms, but not so constant. There are pains, more or less violent, in the lower extremities, with more or less of stiffness both in these and in the upper limbs—trismus —convulsions or paralysis—difficulty of breathing. This last symptom is pretty constant. The convulsions and paralysis may depend on cerebral as well as spinal affections. Tetanus has been ascribed to inflammation of the spinal envelopes; but dissection has not always, though it has sometimes, confirmed this pathology. Authors who have touched on this subject have not, in general, been explicit enough in discriminating inflammation of the spinal marrow from that of its coverings. Dupuytren observed the envelopes alone of the spinal marrow inflamed in a man who died of tetanus. Brera found the spinal marrow itself affected in some cases of this kind.

§ 8. *Myellitis; or, Inflammation of the Medulla Spinalis.* —The *post mortem* appearances, where this part has been the seat of inflammation, are generally a softening of the medullary substance, with more or less of disorganization—sometimes amounting almost to a state of fluidity, yellowish or purulent. Sometimes this disorganization occupies the whole diameter of the spinal marrow, sometimes only half of it. The same may be said of the longitudinal extent of the disease. Many physicians, and among others *M. Recamier*, regard these softenings of the brain and spinal marrow as a degeneration unconnected necessarily with the process of inflammation; but our author, and, indeed, the greater number of continental pathologists, are of a different opinion, and consider the change of structure in question, as the result of inflammatory action more or less acute. It is, perhaps, no valid objection to this doctrine, that inflammation frequently produces an opposite condition, viz. an induration of the spinal marrow. We see, in fact, that phlogosis produces diseased states of other structures of the body, as opposite to each other as those of softening and induration. This induration is not unfrequently accompanied by an augmentation in volume of the part in question; of which Bergamaschi, Portal, and others, have related instances. Esquirol has frequently found the spinal marrow indurated in epileptic patients, and so has the younger Pinel. Our author found this induration and enlargement of the spinal marrow in epileptics, quite independent of any lesion of the brain or its membranes. In a young woman, who became epileptic in the invasion of 1814, and who died in October, 1822, he found the spinal marrow so hard and tough that it could scarcely be torn by the fingers. If the lesion in question be the result of phlogosis, that phlogosis must be, in general, of a very slow kind; as this degeneration is only found in subjects who have long laboured under chronic affections of the nervous system. In respect to *softenings*, however, *they* have been found very often the result of acute forms of inflammation. We shall now proceed to the illustration of *Myellitis* by a selection of cases from the ample store collected by our industrious author.

*Case.* Margaret Marshal, aged 79 years, hitherto enjoying good health, for her time of life, entered the Infirmary on the 26th January, 1822, labouring under symptoms of pulmonary catarrh. A few days afterwards, she complained of violent pain in the head. There was no sensible general disturbance of functions. It was supposed there was slight congestion of the brain, for which aperients were ordered. The cephalalgia continued, and she complained of a sense of formication in one of the arms and one of the legs, the powers of which were, also, diminished, as well as the sense of feeling. At length, complete paralysis of the upper and lower extremities took place, and the woman died comatose. It was prognosticated by M. Rostan and others, that softening of the brain would be found.

*On dissection*, the meninges were found infiltrated, but no softening of the brain could be discovered by the most careful examination. The spine was, therefore, opened; and, in the cervical region, at the upper part, was found a softening of the whole diameter of the medulla, for the space of about an inch and a half. The colour of this part was yellow, and its consistence that of bouillie, without any trace of its natural organization.

*Case.* Jack Prevost, 75 years of age, entered La Pitié on the 12th August, 1822. He reported that he had a fall, in July 1821, on the *left* side of the chest, since which he experienced pain there, and difficulty of breathing. Ten months after the accident, he began to feel a most disagreeable itching in the *left* lower extremity, which he, in vain, attempted to relieve by scratching. Gradually, the itching subsided; and, in proportion, the muscular power of the limb declined, so that, finally, paralysis succeeded the pruritus. The same succession of events took place in the lower extremity of the *right* side. On examination at the hospital, he presented the following symptoms;—slight protuberance at the lower part of the dorsal region of the spine—complete paralysis of the lower extremities—sensibility of the parts greatly diminished, especially in the legs—great torpor of the bowels, the patient not having had a stool for ten days—slight convulsive twitchings of the paralyzed limbs. A seton was inserted in the nucha, and the extract of nux vomica was given, which only increased the spasmodic movements without producing any good. He died on the 31st of the same month.

*Dissection.* In the dorsal region of the spine, a puriform fluid was effused between the bony canal and the meninges, opposite the gibbosity of the spine. The spinal marrow, at the corresponding point, was entirely disorganized, being softened into a substance resembling cream. Immediately above and below this disorganization, the spinal marrow presented its natural structure, except that its vessels were very much injected and its substance was red.

In this case, we cannot but agree with Dr. Ollivier, that the softened portion was the result of an inflammatory action.

We have seen that destruction of the *anterior* roots of the spinal nerves abolished *motion*, and that of the *posterior*, *sensation*, in the parts to which the nerves were distributed. This result of experiments made on animals, is confirmed by the following case in the human subject. The case was communicated to our author by Professor Royer Collard.

*Case of softening of the Anterior Portion of the Spinal Marrow.*—Louis Spreval, a fusileer in the 5th demi-brigade of Veterans, entered the MAISON ROYALE DE CHARENTON, on the 17th October, 1806. No precise information could be collected respecting his complaints anterior to this period. During the first seven or eight years of his residence there, he was taciturn, indolent, and idle. It was difficult to get him from his bed. Few rational answers could be obtained from him on any subject. His gait was unsteady, and his lower extremities tottered as he walked. The motions of his arms were free—pulse slow and feeble—appetite, digestion, and sleep natural. Sometimes he had transient paroxysms of maniacal excitement. By the end of nine years, his lower extremities had become completely paralytic, *yet they preserved their sensibility*. For many years his urine and stools came away involuntarily. His intellectual faculties became quite abolished—he merely ate, drank, and slept. At length, he died, on the 2d March, 1823, in consequence of a bowel complaint.

*Dissection.* Marasmus. Cranium like ivory, and thrice the natural thickness. Dura mater thickened. Arachnoid healthy—as was, also, the pia mater, except where it covers the pons varolii and corpora olivaria, where it was thickened, condensed, and of a blueish colour. On raising the pia mater from the corpora olivaria and pyramidalia, these bodies were found softened and converted into a sort of fluid pulp, which condition obtained along the whole front of the spinal marrow downwards—while it could be traced upwards to the thalami nervorum opticorum, corpora striata, and even into some of the convolutions of the brain. All other parts of the brain were sound in appearance, as was the cerebellum, excepting the commissure of the latter, which was indurated, forming a striking contrast with the neighbouring softened parts. The *anterior roots* of the spinal nerves had lost their natural consistence; while the *posterior* part of the medulla spinalis, and the *nerves* which issue from it, were perfectly sound. It is rare, as our author observes, to find such an exact correspondence between the symptoms during life and the appearances after death. This case is certainly, we think, decisive of the difference of function in the anterior and posterior spinal roots.

It has been remarked that, in almost every instance, the paralysis is on the side *opposite* to that where the injury is seated in the brain—and on the *same side* as the injury or disease of the spinal marrow—proving very fairly that there must be a decussation of the cerebral and cerebellic substance somewhere about the origin of the spinal marrow, and not afterwards. But in the nervous system, the strangest anomalies are sometimes presented, so that we can only make an approach, in most cases, to *probability*, not certainty. Thus, there have been observed unequivocal instances of paralysis on the *same side* as the cerebral lesion, and on the *opposite side* to that of the spinal injury or disease. An example of the *latter* kind (of the *former* some will be found in our Periscope) is quoted by our author from Portal.

*Case.* "A woman had experienced, for many years, smart convulsive twitchings of the left lower extremity at each menstrual period. At 40 years of age, the catamenia ceased, and then the member abovementioned became completely paralytic. Sometime afterwards, convulsive twitchings were felt in the left arm, and the woman died comatose. On inspecting the body and opening the spine, the arachnoid and pia mater covering the spinal marrow, were found inflamed opposite the last dorsal, and first lumbar vertebræ. The spinal marrow itself was reddened and softened on its *right* side, but perfectly sound in appearance on the *left.*" —*Portal Anat. Med. tom.* IV. *p.* 116.

The above is merely an exception to a general rule. It is not more remarkable than a case recorded by M. Jaussen, Surgeon of the Hotel Dieu of Lyons. A young girl of 13 years of age, died after being affected for some time with gibbosity of the spine in the dorsal region. On dissection, two of the vertebræ were found carious, and, at this place, the spinal marrow was flattened considerably for a space of five inches. Its membranes were inflamed, and its nervous pulp reduced to a state of putridity. What was most astonishing was, that four days before her death, the girl had not only moved her limbs, but actually got out of bed without assistance. Neither were the functions of the abdominal or pelvic viscera at all deranged to the last. Such cases are perfectly inexplicable according to our present knowledge, provided no error crept into the observation or recital of them.

There are but few examples on record of *induration* of the spinal marrow. The following is recorded by M. Portal, and it appears to have been the consequence of inflammation.

*Case.* M. De Causan, experienced, at first, a sense of formication in the fingers of the right hand, and, in a little time, of the right foot, which became less sensible, but still possessing the power of motion. These parts became emaciated and cold. These phenomena augmented gradually, extending from the fingers and foot to the fore-arm and leg, the members gradually wasting. The patient went on crutches. The whole of the left side gradually presented the same phenomena as those of the members, the patient being, at length, confined completely to bed, deprived of all power of motion. The respiration and deglutition continued free, nor were the other functions materially disturbed. But the sense of sight and hearing became gradually extinct; and then the other functions were insensibly led into a state of derangement which ultimately ended in death.

*Dissection.* The brain was perfectly sound; but the cervical portion of the spinal marrow was completely indurated, so as to assume a cartilaginous appearance. The membranes covering this indurated portion were red and very much inflamed.

*Case.* In Dr. Abercrombie's paper on spinal affections, we have the case of a man whom he did not see during life, but at whose dissection he was present. He had been subject to a purulent discharge from the left ear, for some years, attended by occasional attacks of severe pain in that side of the head. In April 1817, he was confined part of each day, for a week, by this pain, his appetite being bad and his sleep disturbed; but without much frequency of pulse. About the end of the week, he complained of pain extending down his neck, and, in proportion to this extension of pain, the head became easier. When the pain arrived at the lower portion of the spine, it spread from thence round the body, particularly to the spinous processes of the ilia, accompanied by abdominal uneasiness and pain and difficulty in making water. From the violence of these complaints, his sufferings became such that he could not lie in bed five minutes at a time, being obliged to be constantly walking about his room in extreme agitation, grasping the lower part of his back with both his hands, and gnashing his teeth from intensity of pain. He was sometimes incoherent and unmanageable. In a few days after this he died. No paralytic affection had been observed at any period of the complaint—no dyspnœa—no convulsion.

*Dissection.* No diseased state of brain could be detected. Under the medulla oblongata some gellatinous matter was found, and a considerable quantity of purulent matter flowed from the spinal canal. On laying open the vertebral canal, the spinal marrow, throughout its whole extent, was found covered with purulent matter lying between its membranes. It was rather more abundant at three places than elsewhere—at the upper part of the canal, near the foramen magnum—about the middle of the dorsal vertebræ—and at the top of the sacrum. The substance of the spinal marrow was remarkably soft, and in some places much divided into filaments. All the viscera were sound.*

*Symptoms.* On consulting the various authors who have treated of inflammation of the spinal marrow, we shall find much difficulty in distinguishing the pathognomonic symptoms of this disease. In most cases, inflammation of the substance and of the coverings have been confounded together. According to the author before us, (M. Ollivier) the most constant symptom is exccessively acute and deep-seated pain, accompanied by burning heat in the course of the spine, exasperated by motion. According to Klehss, the pain is also augmented by lying on the back, especially in a feather bed, which admits of curvature of the spine. Pressure does not aggravate the pain. Sense of formication in the limbs, and more or less of involuntary discharge of urine and fæces, generally accompany myellitis. Not very unfrequently we find paralysis beginning below and ascending upwards, till at length the muscles of respiration are paralyzed, and death, by asphyxia, is the result. Sometimes, though more rarely, the paralysis takes a descending course. The pulse is usually frequent and irregular. There are various other phenomena which occasionally attend, as tetanic spasms, aphonia, difficult deglutition, dyspnœa, &c. especially when the disease has acquired a certain degree of intensity. In the midst of this commotion of the vital and animal functions, we find the intellect undisturbed—which is not the case when the arachnoid membrane is the seat of disease.

* Edinburgh Journal, vol. 14.

When treating of the post-mortem appearances it was remarked, that the disease might occupy a part or the whole of the spinal marrow. Our author thinks that, to a certain degree, we may ascertain this circumstance during life by the symptoms. Thus, when the superior portion of the medulla is inflamed, in the neighbourhood of the tuber annulare, there will generally be disturbance of the senses, from the extension of the disease to the encephalon. In these cases we shall have trismus, grinding of the teeth, red tongue, difficult deglutition and speech, irregularity of the respiratory function, vomiting, paralysis of the whole body, and speedy death by asphyxia. The symptoms of hydrophobia are sometimes produced by this state of things. The symptoms attending inflammation of the spinal marrow, as it descends towards the loins, may be easily appreciated, and have been detailed in the cases related.

The above observations relate to *acute* inflammation of the spinal marrow. When it is *chronic*, it is frequently unaccompanied by pain; and paralysis of the limbs, derangement of function in the rectum and bladder, are often the first symptoms which arrest our attention. The three following cases are quoted by Dr. Abercrombie from M. Brera.

"1. A woman, aged 23, who had suffered considerably from syphilis, was seized with a severe quotidian intermittent, which proved very tedious, and resisted all the usual remedies. After some time, it was accompanied by pain in the lumbar region, diarrhœa, tormina, tenesmus, general debility, and emaciation. About three months after the commencement of the fever, she began to be affected with weakness and convulsive motions of the left lower extremity, resembling chorea. In walking, the leg was dragged; and if she attempted by a strong effort a greater degree of motion, it was thrown into convulsive distortions. Soon after, the left arm became affected in the same manner, and there were also convulsive motions of the face and eyes. At this time, the complaints in the bowels continued, but ceased soon after. The other symptoms increased. The difficulty of moving the limbs soon amounted to nearly complete paralysis; and to this were added, difficulty of articulation and diminution of memory. These terminated in loss of speech, coma, and death, which was preceded by general and terrible convulsions. Her death happened rather more than a month after the commencement of the convulsive affection of the leg. On *dissection*, some serous effusion was found in the thorax and in the ventricles of the brain. The spinal marrow was soft and flaccid, and to a considerable extent suppurated. Its investing membrane was in many places covered by a puriform fluid. There was also serous effusion in the spinal canal.

"2. A man, aged 40, was received into the hospital of Crema in the spring of 1804, with no other complaint but general weakness and depression, for which no cause could be assigned. He lay constantly in bed, but complained of no pain; his appetite was good, and he was free from fever. Suspicions being entertained that he was feigning, threats and entreaties were used to induce him to exert himself, but in vain. Meanwhile, from being lean and pale, he became fat and ruddy. Thus he continued through the summer and autumn. As winter approached, he lost his appetite, and became lean and cachectic. In February 1805, he became completely paralytic, both in his legs and arms, and died suddenly in March. On dissection, all was sound in the head, the thorax, and the abdomen. In the spinal canal there was much effusion of bloody sanious fluid, with marks of inflammation and suppuration in the spinal cord, the substance of which was remarkably soft, and tending to dissolution.

"3. A young soldier, who had lately recovered from a petechial fever, was affected with pain in the dorsal vertebræ, difficulty of moving the lower extremities, suppression of urine, involuntary discharge of fæces, general debility, and emaciation. A variety of practice was employed for several months, without relief. The weakness of the lower extremities increased to complete paralysis; and soon after the superior extremities became affected in the same manner. He then lost his speech. After lying a fortnight in this state, completely immoveable and speechless, but in possession of his intellectual faculties, he died suddenly. On *dissection*, there was found no trace of disease in the brain, the thorax, or the abdomen. The spinal cord was inundated by a great quantity of sanious fluid. The cord itself was suppurated, dissolved, and disorganized, at the lower part of the dorsal region. Above this it preserved its natural figure, but was very soft. Its investing membranes, and the periosteum lining the canal of the vertebræ, were destroyed at the part where the cord was so much diseased; the vertebræ and their ligaments were sound.*

Our closing limits oblige us to pass hastily over our author's chapter on the development of morbid tissues in the structure or coverings of the spinal marrow. These have been divided into two classes—namely, into *transformations,* as into bone or cartilage—and *new productions,* as tubercles and hydatids. These four alterations are the only ones which our author has been able to discover in the spinal marrow or membranes, independent of those which we have already treated of in the foregoing pages. They are readily recognized in the dead body—but in the living it would, we think, be impossible to predict their existence. We shall therefore pass on to the last chapter of the work.

§ 9. *Diseases which depend, or are supposed to depend, on Affections of the Spinal Marrow, or its Coverings.* It was

* Edinburgh Journal, vol. xiv. p. 46-7.

the opinion of Alexander of Tralles, that where paralysis affected *only the members*, without the senses of seeing, hearing, or the faculty of speech being troubled, the disease must necessarily have its seat in the spinal marrow. The same notion was entertained by Galen—but it is evidently too exclusive. Hoffman believed, that in *epilepsy* the membranes of the brain are affected, while in *convulsions* the coverings of the spinal marrow were interested. Hence, says he, in the *former* there is loss of sense, while in convulsions this is not necessarily the case. However this may be, we certainly often find disease of the spinal marrow in the bodies of epileptic patients. In ten individuals affected with this complaint, Esquirol found nine with disease of the medulla spinalis. In most of the cases there was softening of the organ in the lumbar region. In one case of chorea, Dr. Guersent found a softening of the lower third of the dorsal region; but our author had an opportunity of examining the body of a child, who had been affected with this complaint, and where the spinal marrow was perfectly healthy.

Not unfrequently, our author observes, we find in the bodies of those who have died of tetanus an inflammation of the spinal marrow, or its envelopes; but, on the other hand, the most rigorous research often fails in discovering any such lesion. Nevertheless, the treatment should always point to this pathology, and such means be employed as tend to remove spinal arachnitis or myellitis. Brera, among others, is an advocate for this doctrine. He has frequently, he avers, found spinal inflammation in the bodies of tetanic patients. One of the cases is curious, and we shall give the particulars of it. A young man received a contusion of the thumb of the right hand, twelve days after which symptoms of tetanus supervened, and the disease was completely established. Being received into the hospital, M. Brera had 120 leeches applied along the spine. The spasms were greatly relieved; but afterwards they became augmented, paralysis took place, and the patient died. On dissection, the spinal marrow was unequivocally inflamed; and, what is curious, the inflammation was confined to the right side of the medulla spinalis—that on which the contusion had occurred.

Our brethren in this country are acquainted with the cases published by Dr. Reid of Dublin, and the plates of the state of the spinal marrow as found by him in tetanus and hydrophobia. There is reason to believe, that the trismus infantum is often dependent on Myellitis. Dr. Thompson of Philadelphia, recognized this state in a considerable number of instances; and Dr. Goëlis of Vienna, has frequently found the same thing in the Foundling Hospital of that city. In many cases of hydrophobia, the spinal marrow has been found inflamed, according to the testimony of Sallin, Clot, Troillet, Matthey, Hufeland, Reid, Dupuy and others. The editor of this Journal found it intensely inflamed, in a case which he dissected a few years ago at Portsmouth, and which was published by Mr. Webster, Surgeon of the 51st regiment, who called him in in consultation. In many cases of fever, the spinal marrow has been found inflamed or its vessels congested, in the experience of M. Chaussier and Dr. Ollivier himself. The same has been corroborated by Dr. Sanders and Dr. Abercrombie of Edinburgh. We have every reason to believe, that a host of derangements in the functions of the thoracic and abdominal viscera are every day produced by slight affections of the spinal marrow, its coverings, or the nerves which emerge from it, whether arising from distortion, or idiopathic inflammation of the part.

We have now brought forward the amplest collection of pathological facts respecting this important and too much neglected portion of the nervous system, which is to be found in any journal, or, indeed, any work in the English language. We have drawn information from many sources besides that of the work before us, and hope that this first article in our new series may prove a useful reference for the junior members, at least, of our profession. The able and industrious author of the work reviewed deserves great praise for the labour which he has bestowed on the subject—and to him we here tender the homage of our respect and esteem.

## Figures and Tables

**Figure f1:**